# A comparative study of an advanced skin imaging system in diagnosing facial pigmentary and inflammatory conditions

**DOI:** 10.1038/s41598-024-63274-7

**Published:** 2024-06-25

**Authors:** Yu-Wen Huang, Walter Arkesteijn, Yi-Jing Lai, Chau Yee Ng

**Affiliations:** 1 Department of Dermatology, Chang Gung Memorial Hospital, Linkou, Taoyuan, Taiwan; 2 Vitiligo Clinic and Pigment Research Center, Chang Gung Memorial Hospital, Linkou, Taoyuan, Taiwan; 3 College of Medicine, Chang Gung University, Taoyuan, Taiwan; 4 Sylton, InnoFaith Beauty Science, Son, The Netherlands; 5 Department of Dermatology and Aesthetic Medicine Center, Jen Ai Hospital, Taichung, Taiwan

**Keywords:** Imaging and sensing, Mechanical engineering, Electronics, photonics and device physics, Optical physics, Techniques and instrumentation

## Abstract

Visual assessment, while the primary method for pigmentation and erythema evaluation in clinical practice, is subjective, time-consuming, and may lead to variability in observations among clinicians. Objective and quantitative techniques are required for a precise evaluation of the disease's severity and the treatment's efficacy. This research examines the precision and utility of a newly developed skin imaging system in assessing pigmentation and erythema. Sixty participants were recruited, and their facial images were analyzed with the new OBSERV 520 x skin imaging system, compared to DERMACATCH for regional analysis and VISIA for full-face examination. The degree of skin pigmentation was clinically graded using the MASI scores evaluated by dermatologists. The data revealed positive correlations between the novel skin imaging system and the two conventional instruments in quantifying pigmentation and erythema, whether in regional or full-face analysis. Furthermore, the new skin imaging system positively correlated with the clinical MASI scores (r = 0.4314, P < 0.01). In contrast, our study found no significant correlation between the traditional system and clinical assessment, indicating a more substantial capacity for hyperpigmentation assessment in the new system. Our study validates the innovative skin imaging system's accuracy in evaluating pigmentation and erythema, demonstrating its feasibility for quantitative evaluation in both clinical and research purposes.

## Introduction

Facial dermatoses often manifest as noticeable skin discoloration, a prevalent concern in dermatology clinics. Among the myriad skin issues, pigmentary disorders are one of the most common problems dermatologists encounter. Dyschromatosis encompasses a broad spectrum of abnormalities in skin pigmentation, including conditions like melasma, solar lentigines, Riehl’s melanosis, and post-inflammatory hyperpigmentation resulting from various dermatoses such as contact dermatitis, eczema, and acne. Additionally, the clinical challenge extends to disorders characterized by the depletion of pigmentation, such as vitiligo and post-inflammatory hypopigmentation. Given the diverse nature of these skin conditions and the possibility of very subtle changes in pigment, making an objective and precise assessment of the severity of the disease becomes paramount in the successful management of pigmentary disorders^1–3^. Dermatologists employ a wide range of noninvasive evaluation techniques to ensure accurate diagnosis, monitor outcomes, and effectively tailor treatments^4,5^. The techniques and mechanisms employed in the literature review included visual assessment, ultraviolet light, photography, polarized light, reflectance spectroscopy, and dermoscopy.

While cosmetology has embraced various skin analysis devices, clinical evaluations of skin discoloration primarily depend on visual assessments such as the MASI scale to evaluate melasma^6^. Although visual assessments underscore the expertise of dermatologists in clinical practice and are indispensable, they are sometimes subjective, time-consuming, and may lead to variability in observations between clinicians^7^. Objective and quantitative techniques, which are non-invasive and convenient, are required for precise evaluation of the disease's severity and the treatment's efficacy. The reliability and reproducibility of the measurements are helpful not only in clinical assessment but also in research purposes. In contemporary medical practice, a diverse array of noninvasive instruments has been introduced, such as dermoscopy^6^, Mexameter®^8^, colorimeter®^9^, DermaSpectrometer®^10^, Chromameter®^10^, VISIA®^11^, and Antera 3D®^12^. These noninvasive instruments help clinicians distinguish and detect color changes that may precede clinical visibility, which means they can be employed as an excellent diagnostic and prognostic tool.

Of all established devices, Dermacatch® (Colorix, Neuchatel, Switzerland) and VISIA® (Canfield, USA) stand out as standard and readily available non-invasive instruments in dermatology departments. Dermacatch® is a colorimeter using entire visible-spectrum reflectance in regional skin analysis. It has demonstrated remarkable specificity and reproducibility in quantification of skin pigmentation and erythema^13^. On the other hand, the VISIA® skin analysis system is the most frequently used device for full-face assessment, which generates a series of photographs using standard, ultraviolet, and cross-polarized lighting for dyspigmentation evaluation.

The VISIA® System was successfully validated by investigating the correlations between measurement methods and capture perspectives^14^. The precision and reproducibility of the system make it an outstanding and reliable tool for dermatology and esthetic practices^15^. OBSERV® 520 x from Sylton, Netherlands, is a newly developed noninvasive instrument for measuring facial skin features, including surface texture, pigmentation, vascularity, wrinkles/lines, volume/lifting, and facial contouring. This study aims to examine the precision and utility of a newly developed skin imaging system in gauging pigmentation and erythema and assess the correlation between instrumental analysis and clinical evaluation.

## Methods

### Instruments

#### Dermacatch®

Constructed with a diode emitting across the entire visible spectrum, the visible-spectrum reflectance colorimeter utilizes a photodetector to quantify melanin and erythema values by assessing globally reflected light. The measurement encompasses a circular area with a diameter of 5.5 mm, equivalent to 24 mm^2^ ^13^, which was employed to analyze the pigmentation and erythema in the area of interest in this study.

#### VISIA®

Equipped with standard, ultraviolet, and cross-polarized lighting at left, front, and right angles of the face, the VISIA® Camera System offers an analysis of eight skin aspects, including spots, wrinkles, skin texture, pores, UV spots, brown spots, red areas, and porphyrins^11^. The system relies on three distinct measurements: the percentile, the feature count, and the absolute score. Among these perspectives, the percentile compares an individual's complexion with those possessing similar skin characteristics. An increase in percentile values signifies an enhancement in complexion, while a decrease in value indicates improvement in other system measurements^14^. In this study, the percentile measurement was employed in the VISIA® system to interpret a baseline assessment of the overall skin analysis in the aspects of spots, UV spots, brown spots, and red areas.

#### OBSERV® 520 x

The newly developed advanced skin imaging system integrates daylight, cross-polarized light, parallel-polarized light, Wood's light, and true UV light to evaluate diverse facial skin features such as surface texture, pigmentation, vascularity, wrinkles/lines, volume/lifting, and facial contouring. Additionally, the device features a patented Face Positioning System, enhancing positioning through visual feedback technology. Images are captured from five angles and analyzed in a holistic facial context and selectively in specific areas. Parameters B1 and B2, measuring pigmentation, encompass the brown spot area (B1), indicating the pigmentation ratio compared to surrounding healthy skin within the selected area, and the brown spot intensity (B2), revealing the severity of pigmentation in the chosen location. Correspondingly, R1 and R2 serve as erythema-related parameters, employing similar principles in their assessment within the system.

### Study population

Sixty participants (4 male and 56 female Asians, aged 45.53 ± 13.31 years) who had been previously diagnosed with facial pigmentary or inflammatory conditions were recruited from September 1, 2022, to September 1, 2023, in the Department of Dermatology at Chang Gung Memorial Hospital, one of the tertiary medical centers in Taiwan. The facial conditions of the volunteers were evaluated and confirmed by two certified dermatologists. The facial pigmentary conditions include melasma, freckles, lentigo, post-inflammatory hyperpigmentation, and vitiligo; inflammatory conditions include rosacea, acnes, and telangiectasia. Among 60 participants, 36 had pigmentary conditions, 16 had inflammatory conditions, and 8 had both pigmentary and inflammatory disorders. Individuals under 20 and women who were pregnant or lactating were excluded from the study. Additionally, individuals lacking the capacity to comprehend the research protocol or actively engage in the study were excluded. Subjects were enrolled in this study after providing written informed consent, and all subjects and/or their legal guardian(s) consented to the publication of identifying information/images in an online open-access publication. We confirmed that all experiments were performed in accordance with relevant guidelines and regulations.

### Measurements

Participants were instructed to cleanse their faces 20 min before the tests. All measurements from VISIA® and OBSERV® 520 x were conducted under controlled dark conditions in the same room. Facial images from various angles were captured using VISIA® and OBSERV® 520 x respectively. The facial features of all patients were examined under uniform conditions, including consistent lighting, background, positioning, and observing distance. For regional analysis, two dermatologists were tasked with identifying the most severe spot within the lesion area, utilizing Dermacatch® and focal area analysis mode of OBSERV® 520 x to assess both pigmentation and erythema in selected area.

### Grading by the dermatologist

In the visual assessment of the study, two certified dermatologists meticulously evaluated the skin conditions of the participants concerning pigmented areas immediately following the acquisition of facial images using three distinct devices. The degree of skin pigmentation was systematically graded by employing the Melasma Area and Severity Index (MASI) score^6^, indicating a clinical analysis of the participants' overall facial skin pigmentation levels. The final MASI score was determined by calculating the average of the scores provided by the two dermatologists.

### Statistical analysis

The outcomes derived from OBSERV® 520 x were juxtaposed with readings obtained from two well-established devices: Dermacatch® for regional analysis and VISIA® for comprehensively examining the entire face. Furthermore, to assess the practical utility of the novel skin imaging system, the results were also compared against clinical MASI scores. Correlations between Dermacatch®, VISIA®, and OBSERV® 520 x were determined by calculating the Pearson correlation coefficient between the readings. All the data were performed by SPSS 22.0 software (IBM Corporation) and GraphPad Prism 9 software (Dotmatics). URL links are https://www.ibm.com/support/pages/spss-statistics-220-available-download and https://www.graphpad.com/updates. Results were considered statistically significant at a p-value less than 0.05.

Notably, the percentile served as the measurement in the VISIA® system in this study, where an increase indicated improvement. However, in the OBSERV® 520 x parameters and clinical MASI score, an increase denoted a deterioration in pigmentation. Consequently, correlation coefficient values exhibited negative numbers compared to the VISIA® system. These negative numbers were disregarded for ease of interpretation, and only the absolute values of the coefficients were considered for comparison to illustrate the degree of correlation.

## Ethics declarations

Reviewed and approved by Chang Gung Medical Foundation Institutional Review Board; approval #202201032B0.

## Results

### Instrumental differences between the three instruments

**Figure 1:**
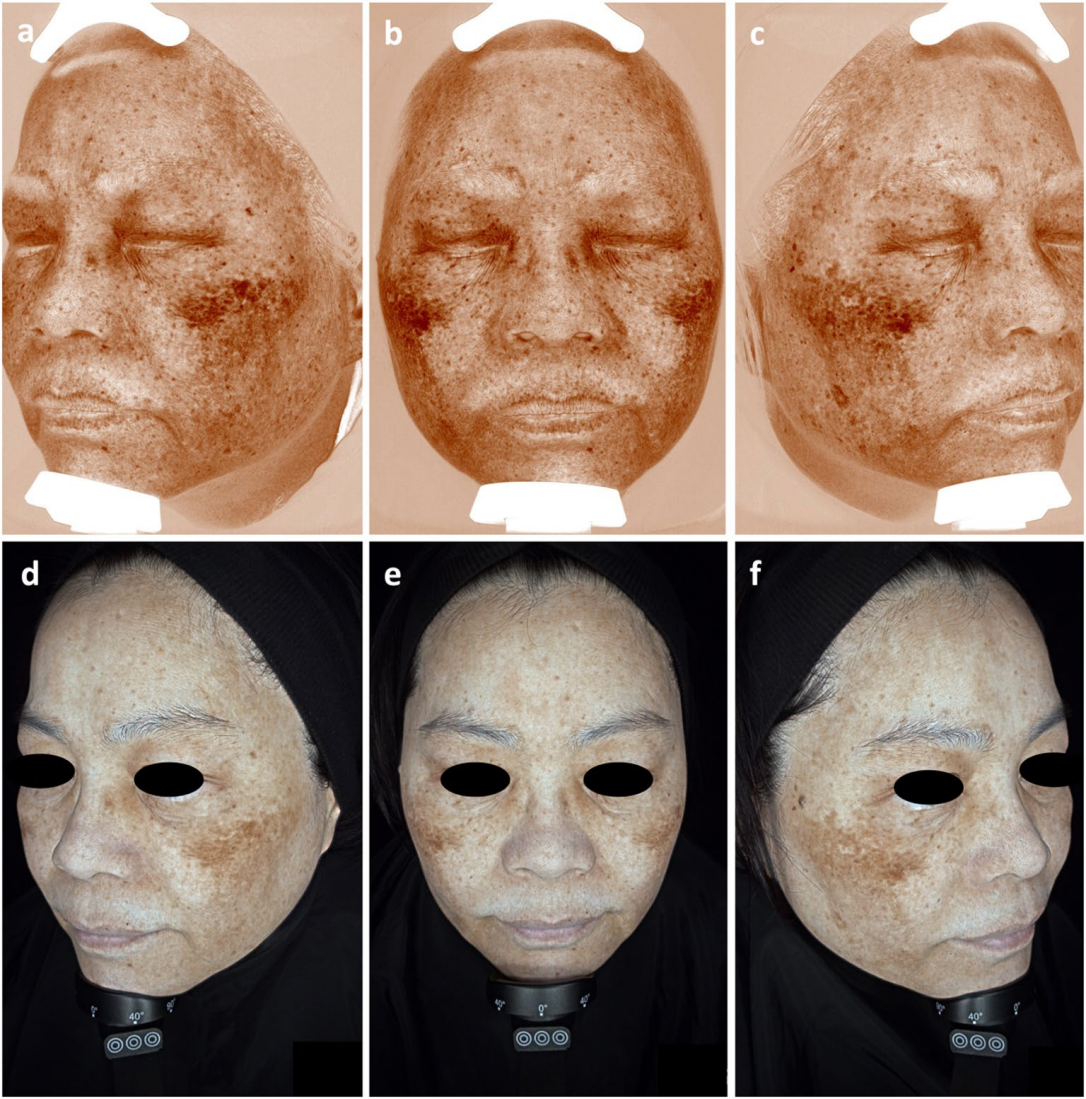
Images of pigmented area captured by V-system and O-system. **a**–**c** show the brown spots mode of the V-system. The melanin functions as an indicative marker for brown discoloration. The system assesses pigmented lesions by quantifying melanin content, employing cross-polarized illumination to generate images that facilitate the detection of deeply deposited melanin. **d**–**f** illustrate pigmentation evaluation of the O-system. Pigmentation in this system was measured using parameters B1 and B2, which include the brown spot area (B1) indicating the pigmentation ratio compared to surrounding healthy skin within the selected area, and the brown spot intensity (B2) revealing the severity of pigmentation in the chosen location. The analysis in the new system is either full-face or focused on a selected area, whereas the V-system only assesses the overall condition of the entire face.

Pigmented lesion indices were recorded and measured using the D-colorimeter, the V-system (Fig. 1a–c), and the O-system (Fig. 1d–f). The analysis in the new sysetem can be either full-face or focused on a selected area, while the D-colorimeter is limited to evaluating local areas, and the V-system only assesses the overall condition of the entire face. Furthermore, unlike the V-system, the novel system incorporates additional light sources such as Wood's light and parallel polarization. It conducts image analysis from more perspectives within a reduced timeframe. Additionally, the equipment with a patented Face Positioning System is unique from the old ones. A brief comparison of the three instruments is summarized in Table 1.

**Table 1:** Brief comparison of three instruments.

	D-colorimeter	V-system	O-system
Light sources	A diode emitting in the full visible spectrum	Standard, UV, cross-polarized lights	Daylight, Cross polarized, Parallel polarized, Wood’s, True UV lights
Color channel	Entire visible-spectrum reflectance	RGB	Solid state LED
Angles	NA	3 angles (front, left, right)	5 angles (0, L40, L90, R40, R90)
Analyzed indices	Pigmentation and erythema	Spots, wrinkles, textures, pores, UV spots, brown spots, red areas, porphyrins	Surface texture, pigmentation, vascularity, wrinkles/lines, volume/lifting, facial contouring
Values	Melanin and erythema values	Feature counts, absolute scores, percentiles	R1, R2, B1, B2
Skin pigmentation-related parameters	Melanin value	Spots, UV spots, brown spots	B1, B2
Skin erythema-related parameters	Erythema value	Red areas	R1, R2
Numbers of images	1	24	8
Requiring time	Instant	2 min	10 s
Analysis area	Focal, measured area with a disk of 5.5 mm in diameter (24 mm^2^)	Full face	Full face and focal area
Other equipment			Patented Face Positioning System (FPS)

D-colorimeter, Dermacatch®; V-system, VISIA®; O-system, OBSERV® 520 x; NA, not applicable; PC, personal computer; RGB, red–green–blue; UV, ultraviolet.

### Correlations of the facial features of hyperpigmentation between the new skin image system and the two conventional instruments

**Figure 2:**
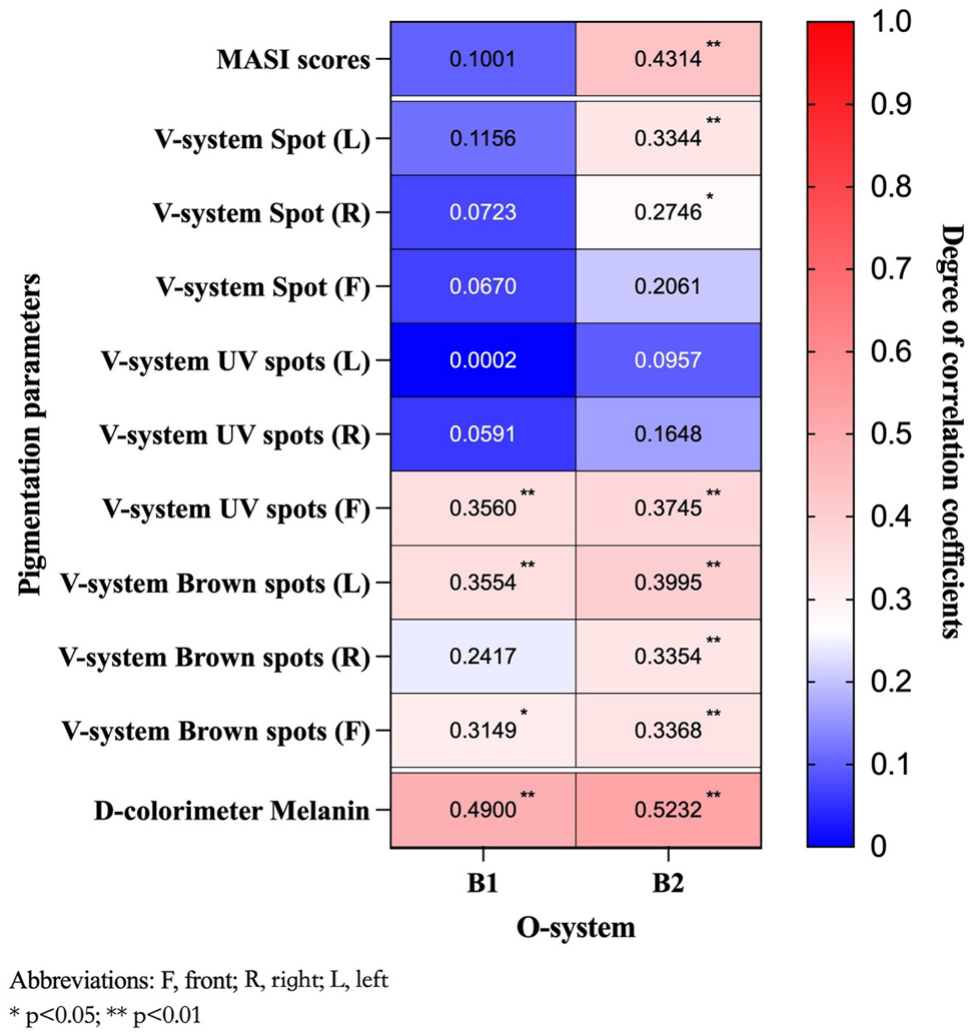
Heatmap of the correlation coefficients between the new skin image system and the two traditional devices/ clinical visual scores in pigmentation assessment. A higher correlation value of the two devices corresponds to a value closer to 1. Asterisks indicate statistically significant results.

In regional analysis, pigmentation-related parameters B1 and B2, measured by the new skin imaging system, showed significant moderate correlations with melanin values in the D-colorimeter (r = 0.4900, P < 0.01; r = 0.5232, P < 0.01, respectively). For full-face examination, the new skin imaging system exhibited significant correlations with the brown spots mode of the traditional V-system at different face angles (r = 0.3149–0.3995, P < 0.05). Only B1 on the right side of the new system showed no significant difference (P > 0.05). Furthermore, relatively weaker or no correlations were found between the new image system and other pigmentation-related parameters measured by the V-system, such as spots and UV spots. The correlation coefficients between the new skin imaging system and the two traditional devices in pigmentation assessment were summarized in a heatmap, as shown in Fig. 2.

### Correlations between parameters analyzed by the instruments and the visual scores graded by dermatologists

In comparing the results from the instruments to the subjective evaluation by physicians, parameter B2 in the new skin imaging system positively correlated with the clinical MASI scores (r = 0.4314, P < 0.01), while no significant correlation was found between the traditional system and clinical assessment (Table 2 and Fig. 2).

**Table 2:** Correlations of visual grading and pigmentation-related parameters measured by V-system and O-system.

	Parameter	r	P
Visual grading	MASI vs V-system Spots	0.2129	0.1741
	MASI vs V-system UV spots	0.1210	0.4571
	MASI vs V-system Brown spots	0.1983	0.2199
	MASI vs O-system B1	0.1001	0.5388
	MASI vs O-system B2	**0.4314**	0.0054**

*r*, the Pearson correlation coefficient; P, p-value.

### Correlations of the facial redness between the new skin image system and the two conventional instruments

**Figure 3:**
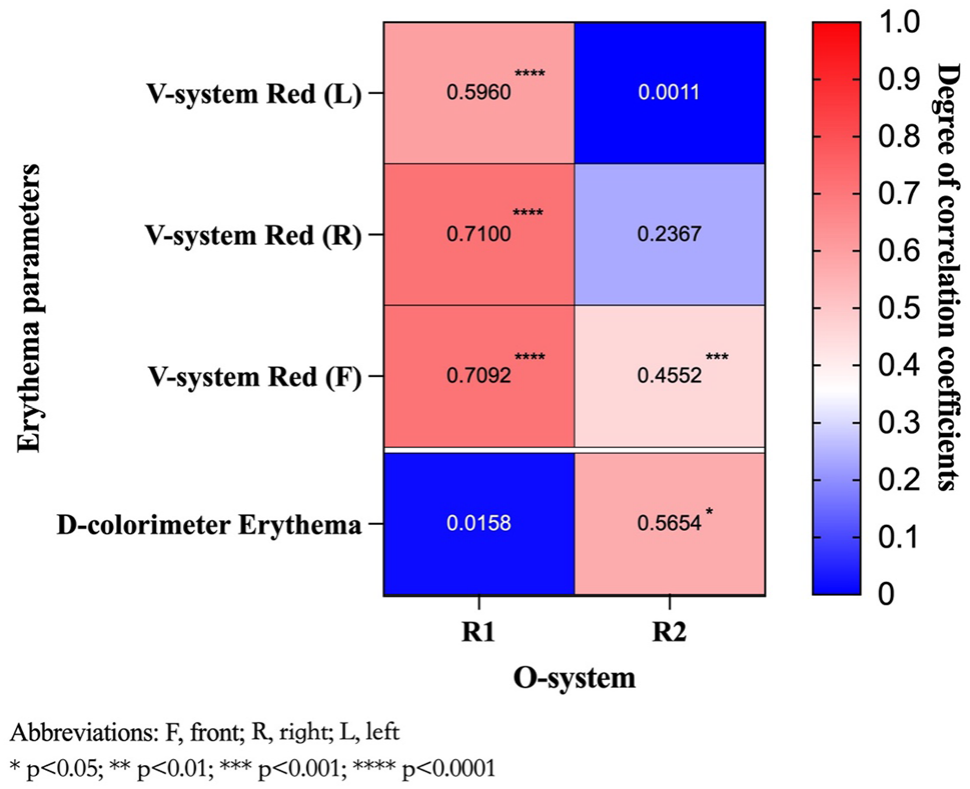
Heatmap of the correlation coefficients between the new skin image system and the two traditional devices in redness assessment. A higher correlation value of the two devices corresponds to a value closer to 1. Asterisks indicate statistically significant results.

Illustrated in Fig. 3, the analysis of facial erythema was conducted and compared using the three devices in our study. The new skin imaging system demonstrated moderate to strong correlations with the two traditional instruments, whether in regional or the entire face analysis.

## Discussion

Melanin, hemoglobin, bilirubin, and carotene levels within the skin influence the predominant determinants of skin color. Skin hyperpigmentation mainly arises from alterations in the production and distribution of melanin^16^. Unified criteria and well-controlled conditions for the patients are required for a reliable evaluation of skin discoloration. However, in clinical practice, the assessment conducted by a physician may be influenced by various factors beyond non-standardized evaluation criteria, including differences in illuminations, diverse clinical experiences, subjective elements, and patient feedback^17^, which often leads to variability in observations. Moreover, it is sometimes challenging to determine skin conditions by the human eye when the change in skin is minimal. Therefore, the incorporation of skin imaging system does offer a benchmark that could assist clinicians in quantifying the severity of the skin color change, formulating personalized therapeutic approaches, and monitoring treatment responses.

Objective and non-invasive devices with validation serve as valuable diagnostic tools in clinical practice. In the present study, we compare the newly developed skin imaging system with two well-established instruments. Both have been clinically applied to assess various skin conditions and diseases, including vitiligo^18^, rosacea^19^, facial pores^20^, and facial photoaging^21^. Furthermore, their effectiveness in evaluating treatment responses has also been validated in the literature^22,23^.

In our study results, the new skin imaging system exhibited consistency with the two well-known devices in assessing pigmentation and erythema, whether in regional or full-face evaluation. This highlights its potential application value as an alternative tool. Besides, of the two pigmentation-related parameters measured by the new skin imaging system, parameter B2 always exhibited a stronger correlation than B1 when compared with the two traditional devices and clinical MASI scores, regardless of whether significance was observed or not. This suggests that the new system is more accurate in assessing the severity of pigmentation than the pigmentation ratio in the selected area. The reading of B2 may provide a more representative analysis of pigmentation by the new skin imaging system.

When compared with visual grading evaluation, the new skin imaging system not only demonstrated a significant correlation with MASI scores but also exhibited a stronger capacity for hyperpigmentation assessment than the traditional V-system. Therefore, we consider the new skin imaging system more precise than the traditional device in measuring skin pigmentation. In the conventional devices, only the severity of whole face pigmentation and erythema is measured among the parameters. The advantage of the new O-system is that the parameters of B2 and R2 not only measure the severity of pigmentation and erythema but also include B1 and R1 as measurements of involvement ratio compared to normal skin area. Although the current O-system did not incorporate MASI score as the outcome measurement, by simultaneously considering B1 and B2, we can quantitatively interpret the severity and involvement ratio of pigmentation across the entire face, similar to the clinical value of the MASI score. Consequently, the new skin imaging system offers better complexity and practicality than the older ones. Moreover, whereas the two conventional devices are limited to assessing either specific regions or the entire face independently, the newly developed skin imaging system demonstrates the capability for comprehensive analyses, encompassing both full-face and selected area assessments within the same image set. Hence, we were able to analyze either the overall skin condition or a specific regional lesion using different modes of the new system, potentially demonstrating beneficial effects in both dermatology and cosmetology. Apart from assessing pigmentation, the new system can also evaluate depigmentation by incorporating Wood's light source, which is lacking in the conventional systems. On the other hand, the new skin imaging system captures 8 high resolution pictures through 5 set angles in 10 s, which provides a faster and more convenient reference than the previous devices for clinical analysis and could facilitate large-scale population studies for research purposes. The above advantages demonstrate that the practicality and capabilities of the new system surpass those of the older devices. Nevertheless, the new machine exhibits a higher cost than the old ones and lacks the portability found in other devices designed for regional assessment.

While objective determination of skin color becomes essential in managing pigmentary disorders, the readings of available devices can be influenced by factors such as hair color and length, excessive stretching of the skin, incomplete contact of the device, and the pressure applied by the device to the skin^24^. These variables have the potential to induce noteworthy alterations in reflectance, consequently affecting the obtained readings. Notably, the advanced skin imaging system is equipped with a patented Face Positioning System, designed to facilitate the positioning of patients through the utilization of visual feedback technology. This exclusive feature of the new system contributes to the reduction of errors stemming from mechanical factors.

Some limitations need to be considered in the present study. Firstly, the results of the new device were solely compared with the percentile measurement in the traditional V-system, rather than other metrics. The study would show greater strength if additional measurements, such as feature count and absolute score, exhibited consistent linear correlations with the new device. Secondly, despite implementing a standardized protocol and maintaining a controlled environment for all measurements in the study, the assurance of precisely identical temperature and humidity conditions for each participant during the examination is not guaranteed. Ensuring unified evaluation criteria and strictly controlled conditions for patients is crucial for establishing reliable comparisons, as even minor variations in the examination process may contribute to result variability. On the other hand, race and skin type were not specified or subclassified in the study. The potential influence of underlying skin color on the precision of the analysis from the devices remains uncertain. Additionally, all measurements in the present study were conducted solely at a fixed time point, without comparing correlations across different timelines. Future studies will be needed to validate the accuracy of dynamic changes across different timelines and to compare them with both the conventional system and the expertise of professionals. Finally, apart from evaluation of pigmentation, facial erythema underwent analysis using the three devices in the study, unveiling moderate to strong correlations between the new system and the two traditional instruments. Nonetheless, a comparison of the new device with clinical evaluation of erythema was lacking.

Accordingly, further studies are needed for the novel skin imaging system to verify the evaluation ability of redness in comparison with clinical expertise, and the evaluative capacity of other parameters, including surface texture, wrinkles, and volume. It has been suggested that the measurement of melanin may be falsely biased by fluctuations in skin erythema and vice versa due to a partial overlap in wavelengths^13^. Consequently, in the future research, diverse underlying skin conditions may be compared when assessing pigmentation and erythema from the new device, aiming to verify its capacity to discriminate between cutaneous melanin and erythema. Moreover, incorporating multiple time points into the analysis would offer a more comprehensive understanding of the system's effectiveness in tracking skin evolution and treatment responses, thereby enhancing the system's clinical utility.

## Conclusion

In conclusion, our study validates the accuracy of the newly developed skin imaging system in evaluating pigmentation and erythema. Compared to previous devices, the novel system demonstrated a higher correlation with visual scores evaluated by dermatologists and exhibited numerous advantages in facilitating precise diagnosis and treatment. The advanced skin imaging system is feasible for quantitative evaluation in both clinical and research settings, thereby not only reinforcing professionalism but also emphasizing a commitment to dermatological care.

## Data Availability

Data is available on request from the authors, and individuals seeking access may communicate with the corresponding author for further assistance.
